# Supplementary material for: MetaRibo-Seq measures translation in microbiomes
Source: Nat Commun. 2020 Jun 29;11:3268. doi: 10.1038/s41467-020-17081-z (PMC7324362; doi:10.1038/s41467-020-17081-z)
Supplement: Supplementary file 10 — Supplementary Data 7 [file 41467_2020_17081_MOESM10_ESM.zip › File2/Confidence_High_Taxonomy/44071_out.krona.html]

Javascript must be enabled to view this page.

members
magnitude
magnitudeUnassigned
count
unassigned
taxon
rank

44071\_out

2

1
2
superkingdom

1
201174
phylum

class
1760
1

1
85011
order

family
2062
1


SRS075341\_contig\_number\_12535
1
genus
1883

superkingdom
2759
1

kingdom
4751
1

1
451864
subkingdom

4890
phylum
1

147538
subphylum
1

class
147550
1

1
222544
subclass

1
5151
order

family
5152
1

5153
genus

SRS058070\_contig\_number\_23194
1
